# Barrier-to-Autointegration Factor 1: a key regulator of nuclear envelope integrity, genome stability, and disease progression

**DOI:** 10.3389/fimmu.2026.1870828

**Published:** 2026-07-23

**Authors:** Pengyu Shi, Shanpeng Wang, Zixuan Zang, Dalin Wang, Cuiyuan Yang, Jinghan Tao, Jing Liu, Jinpeng Sun, Wenzhi Shen, Rong Wang, Min Sun

**Affiliations:** 1 College of Clinical Medicine, Jining Medical University, Jining, Shandong, China; 2 College of Stomatology, Jining Medical University, Jining, Shandong, China; 3 College of Integrative Chinese and Western Medicine, Jining Medical University, Shandong, Jining, China; 4 Shandong Provincial Precision Medicine Laboratory for Chronic Non-communicable Diseases, Institute of Precision Medicine, Jining Medical University, Jining, China

**Keywords:** BANF1, DNA binding, DNA damage repair, immune regulation, mitosis, nuclear envelope rupture, tumorigenesis

## Abstract

Barrier-to-Autointegration Factor 1 (BANF1) is a self-associating protein encoded within the q13.1 locus of chromosome 11. It is integral to multiple cellular processes, including cell cycle regulation, chromatin organization, gene expression modulation, nuclear envelope (NE) repair, DNA damage repair (DDR), innate immune function, and viral infection control. While prior investigations have provided preliminary phenotypic and bioinformatic characterizations of BANF1 across various disease states, comprehensive analyses detailing its functional roles and mechanistic underpinnings in diverse pathological contexts remain scarce. In cancer, BANF1 is frequently upregulated, with cancer cells leveraging its functions to preserve NE integrity, inhibit activation of the cyclic GMP-AMP synthase-stimulator of interferon genes (cGAS-STING) immune pathway, and facilitate epithelial-mesenchymal transition (EMT). These activities contribute to enhanced genomic stability and promote tumor cell proliferation and migration, thereby conferring oncogenic properties. In neurodegenerative diseases, BANF1 is implicated in disease pathogenesis, exemplified by its mediation of glutamate-induced oxidative stress and apoptosis in neuronal cells, as observed in Alzheimer’s disease (AD). Additionally, rare mutations in BANF1 are associated with hereditary conditions: the recessive Ala12Thr (A12T) variant causes Néstor-Guillermo progeria syndrome (NGPS), whereas the dominant Gly16Arg (G16R) mutation results in dominant motor neuronopathy. This review further synthesizes recent progress in exploring BANF1 as a therapeutic target, encompassing the development of small-molecule inhibitors, immunomodulatory approaches, and its potential applications in cancer treatment. Overall, this article provides a comprehensive overview of BANF1’s structural features, cellular functions, and involvement in disease initiation and progression, with particular emphasis on its expression profiles in malignancies and the therapeutic promise of BANF1-directed interventions.

## 1 Introduction

**Figure 1:**
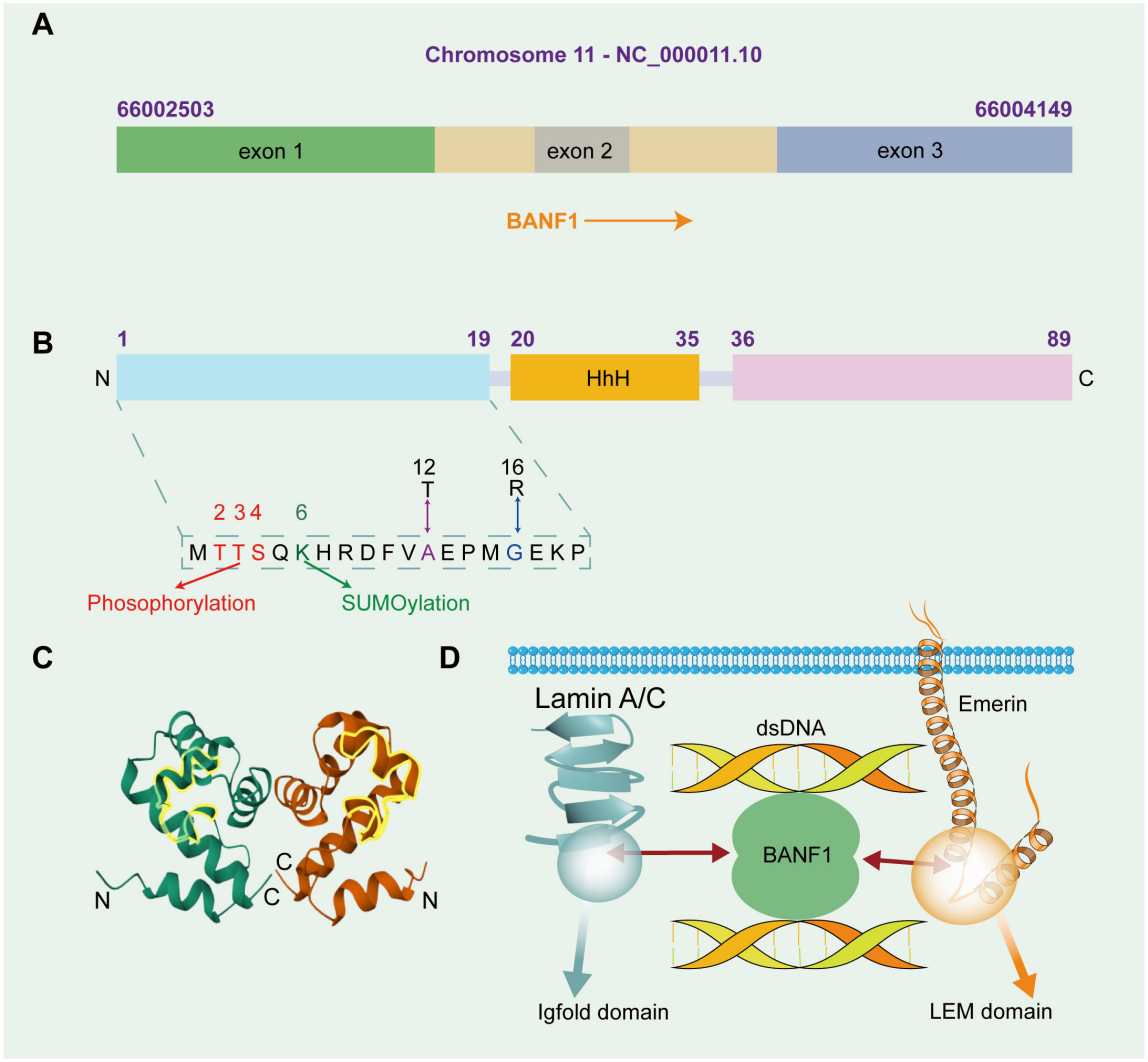
Basic information and structure of BANF1. **(A)** Location of the BANF1 gene on the human chromosome (NCBI database). **(B)** Information on the core domain of the BANF1 protein (UniProt database) and specific amino acid sites: HhH domain spans amino acids 20-35; T2, T3, and S4 are prone to phosphorylation; K6 is susceptible to SUMOylation. The A12T causes NGPS, and the G16R leads to dominant motor neuron disease. **(C)** Schematic of the three-dimensional structure of the BANF1 dimer. The monomers are shown in green and orange, respectively. The yellow fluorescently labeled region indicates the HhH motif. **(D)** Diagram illustrating the binding of the BANF1 dimer to dsDNA, emerin, and lamin A/C.

Barrier-to-Autointegration Factor 1 (BANF1) is an 89-amino acid protein encoded by human chromosome 11q13.1 (**Figure 1A**). It can form a homodimer and was initially identified as a host factor that inhibits autointegration of Moloney murine leukemia virus (MMLV) (1). BANF1 is widely involved in various critical cellular biological processes, including cell cycle progression, regulation of gene expression, chromatin organization, nuclear envelope (NE) rupture repair, DNA damage repair (DDR), innate immune response, and defense against viral infection (2–5). The loss of BANF1 directly causes mitotic defects in nematodes and Drosophila melanogaster, ultimately leading to embryonic lethality (6, 7).

In recent years, the abnormal expression and dysfunction of BANF1 have garnered significant attention in various diseases. In tumors, BANF1 is significantly overexpressed in multiple malignancies, including colorectal cancer (CRC), gastric cancer (GC), esophageal squamous cell carcinoma (ESCC), triple-negative breast cancer (TNBC), liver hepatocellular carcinoma (LIHC), and head and neck squamous cell carcinoma (HNSCC). Its high expression is closely associated with poor prognosis in patients. BANF1 drives tumor progression through multiple mechanisms, such as promoting tumor cell proliferation, migration, and invasion; inducing epithelial-mesenchymal transition (EMT); regulating DDR; and remodeling the tumor microenvironment (TME) (8–13). In other human diseases, point mutations in the N-terminal region of the BANF1 gene can lead to different clinical phenotypes: the Ala12Thr (A12T) recessive mutation causes Néstor-Guillermo progeria syndrome (NGPS), while the Gly16Arg (G16R) dominant mutation results in dominant motor neuronopathy (14, 15). This region is also a critical site for post-translational modifications such as phosphorylation and SUMOylation, which finely regulate BANF1’s binding activity and function (16, 17) (**Figure 1B**). Additionally, in neurodegenerative diseases like Alzheimer’s disease (AD), upregulation of BANF1 expression may participate in regulating neuronal apoptosis (18). Therefore, the pathological roles of BANF1 exhibit dual characteristics of both “quantitative” and “qualitative” changes, manifesting not only as abnormal upregulation of expression levels but also involving protein structural alterations caused by gene mutations.

Although existing studies have preliminarily revealed the expression profile changes of BANF1 in various tumors and its association with clinicopathological features, research on its specific molecular mechanisms, targeted intervention strategies, and therapeutic potential of BANF1 remain inadequately explored. For example, key issues such as the fine regulatory network of BANF1 in TME remodeling, the role of its phosphorylation modifications in tumor drug resistance, and the progress in developing small-molecule inhibitors targeting BANF1 still require in-depth exploration. This review aims to consolidate the multifunctional roles of BANF1 within the framework of NE homeostasis, systematically elucidate its structural characteristics, biochemical properties, and cellular functions, with a focus on analyzing BANF1’s expression profile and mechanisms in tumors. It also discusses therapeutic strategies targeting BANF1 and its potential value as a biomarker, providing a theoretical basis for the precise diagnosis and treatment of BANF1-related diseases.

## 2 Structure and biochemical properties

### 2.1 Interacting proteins and key sites

BANF1 functions as a homodimer, with each monomer non-specifically binding double-stranded DNA (dsDNA) through a helix-hairpin-helix (HhH) motif, including both genomic DNA and exogenous DNA (19–22). The dimer interface forms a distinctive groove that serves as the primary binding site for LAP-Emerin-MAN1 (LEM) domain proteins, such as the LAP2 family, emerin, MAN1, LEM-2, and others (23–29) (**Figure 1C**). Notably, BANF1 located on the NE is highly mobile, whereas emerin is relatively fixed, suggesting that their interaction may exhibit a dynamic “touch-and-go” mode (30). Additionally, BANF1 can also directly but weakly interact with A-type lamins (mainly lamin A/C) by binding to their immunoglobulin-like fold (Igfold) domain (23). During nuclear assembly, BANF1 dimers bind DNA and simultaneously bridge LEM-domain proteins (emerin, LAP2α) with lamin A/C, thereby indirectly promoting their interaction (23, 26, 31, 32) (**Figure 1D**). This interaction is attenuated by BANF1 phosphorylation (33).

BANF1 is distributed throughout both the cytoplasm and the nucleoplasm, with its localization varying according to the cellular state, age, and induction conditions (5, 16, 34). During the interphase of mitosis, BANF1 transport between the cytoplasm and nuclear compartments is minimal, potentially contributing to nuclear structural homeostasis (34, 35). In early mitosis, as the NE disassembles, phosphorylated BANF1 (pBANF1) diffuses into the cytoplasm. Conversely, during mitotic exit, BANF1 undergoes dephosphorylation and relocalizes to the nucleus, participating in NE reassembly (36, 37). Additionally, a BAF-like protein (BAF-L), encoded by the BANF2 gene, shares 53% similarity with BANF1 and forms heterodimers with it. Unlike the ubiquitously expressed BANF1, BAF-L is highly expressed specifically in the pancreas and testes and exhibits weaker DNA-binding capacity (38). Preliminary studies suggest that BAF-L may participate in chromatin remodeling during spermatogenesis (histone/protamine exchange), but its precise function and relationship with BANF1 remain to be elucidated (39, 40).

### 2.2 Phosphorylation and SUMOylation of BANF1

**Figure 2:**
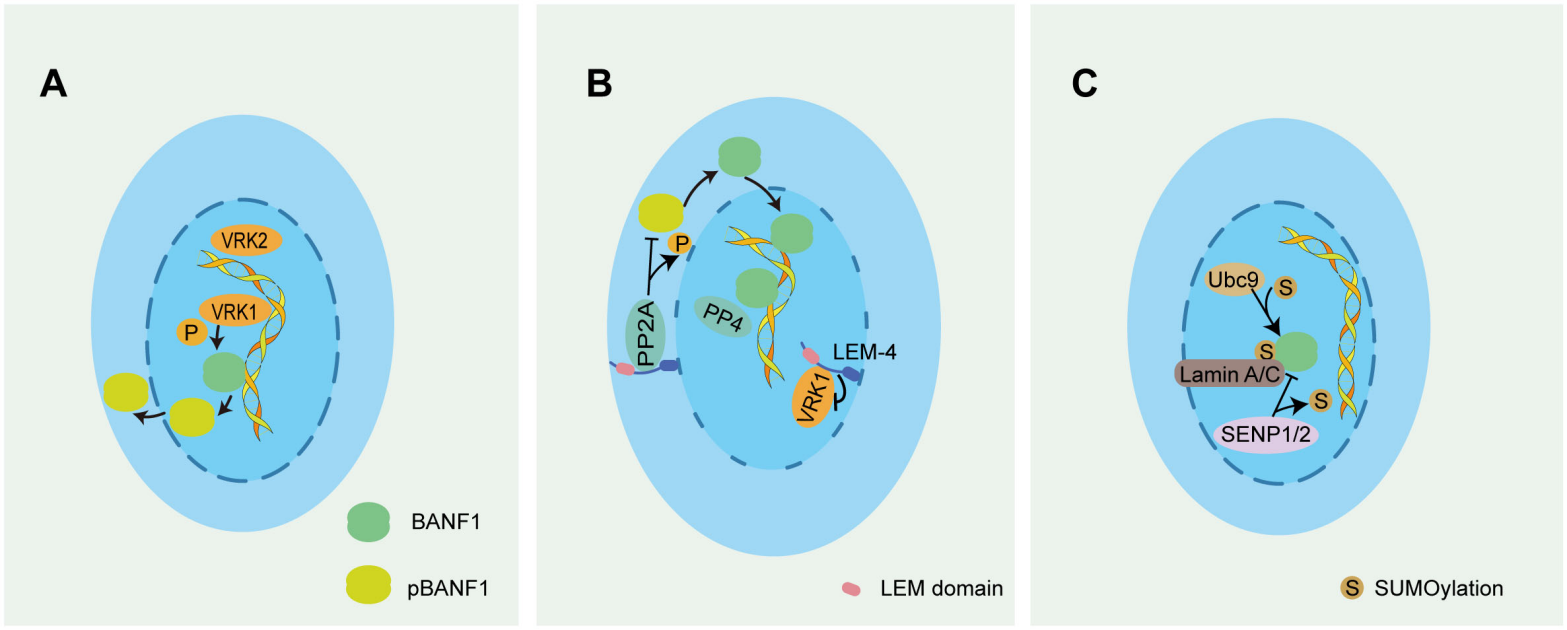
Post-translational modifications of BANF1. **(A)** Phosphorylation mediated by VRK1 and VRK2 during early mitosis. **(B)** Dephosphorylation mediated by PP4, LEM-4, and PP2A during mitotic exit. **(C)** The cycle of SUMOylation and deSUMOylation.

The vaccinia-related kinases (VRKs), including VRK1 and VRK2, phosphorylate the N-terminal residues Ser-4 (preferentially), Thr-2, and Thr-3 of BANF1 during early mitosis, inducing its conformational closure and dissociation of the DNA bridge, thereby facilitating mitotic progression (36, 41–44) (**Figure 2A**). Importantly, the reduced DNA-binding affinity of BANF1 is not caused by direct interference from the phosphate group itself but results from phosphorylation-induced restriction of N-terminal flexibility and alterations in secondary structure (16, 41, 45). Knockdown of VRK1 significantly inhibits BANF1 phosphorylation, causing its abnormal retention on chromatin during early mitosis and subsequently impairing chromatin compaction and NE dynamics (44, 46, 47). This regulatory pathway is evolutionarily conserved; for example, in meiosis of Caenorhabditis elegans, VRK1 phosphorylates BANF1 at Ser-4 to regulate chromatin state. The absence of VRK1 leads to abnormal chromosome anchoring to the NE and delayed progression of cell division (48–50).

During post-mitotic NE remodeling, protein phosphatase 4 (PP4) and protein phosphatase 2A (PP2A) dephosphorylate BANF1, facilitating its role in NE reconstruction (51, 52). LEM-4 (Ankle2) plays a pivotal regulatory role: it binds to VRK1 to inhibit its kinase activity (38) and, as a regulatory subunit of PP2A, locally enhances dephosphorylation within the NE (37, 51, 53) (**Figure 2B**). Knockdown or deletion of LEM-4 or PP2A results in sustained hyperphosphorylation of BANF1 during mitosis, impairing normal NE reorganization (54, 55).

Beyond phosphorylation, BANF1 undergoes SUMOylation at the lysine 6 (K6) residue. This modification, catalyzed by the ubiquitin-conjugating enzyme 9 (Ubc9) and reversed by sentrin-specific proteases (SENP1 and SENP2), is essential for BANF1’s stable binding to lamin A/C and its proper localization to the NE (**Figure 2C**). A BANF1 K6R mutation (lysine changed to arginine) results in abnormal NE assembly and DNA replication defects (17). These findings further demonstrate that SUMOylation constitutes a critical regulatory layer governing BANF1’s normal function.

## 3 Cellular function

### 3.1 BANF1 in NE disassembly and reassembly during mitosis

**Figure 3:**
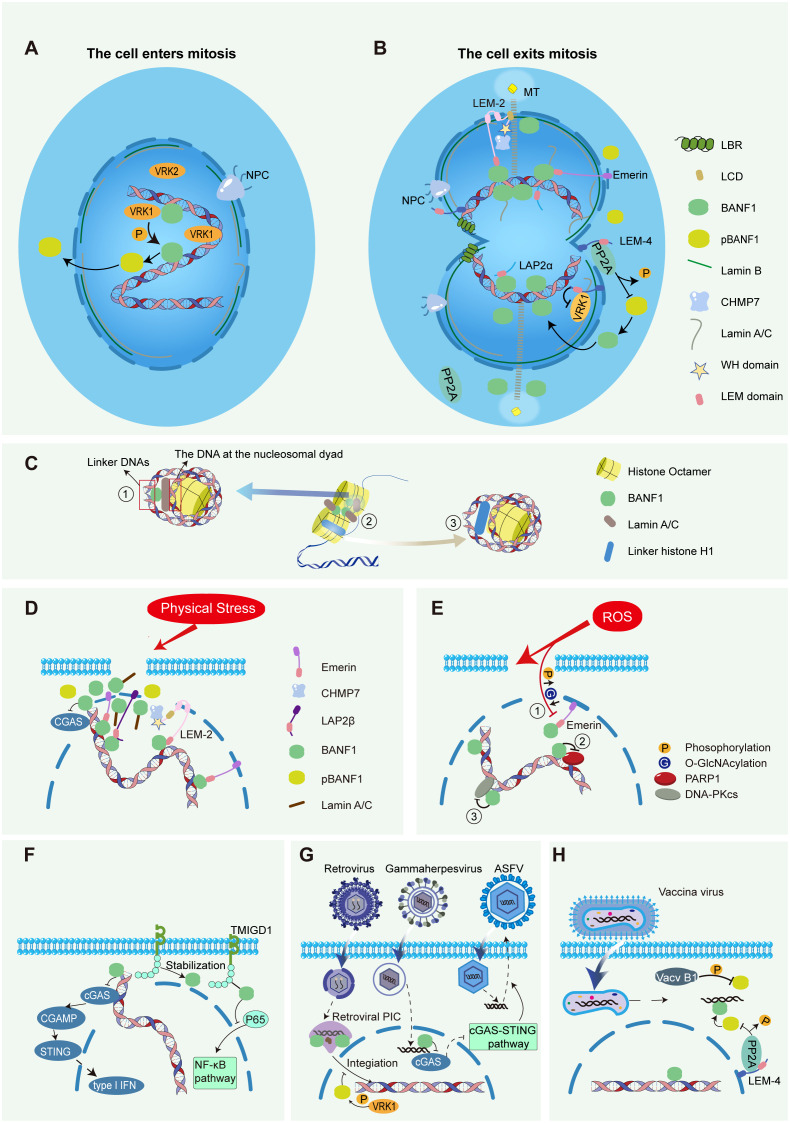
Cellular functions of BANF1. **(A)** Phosphorylation of BANF1 during early mitosis promotes NE breakdown. **(B)** Dephosphorylation of BANF1 and the ESCRT-III complex during mitotic exit mediates NE remodeling. **(C)** Distinct roles of BANF1 in chromosome organization: 1. In the absence of linker histone H1, BANF1 and lamin A/C are located at the nucleosomal dyad and linker DNAs. 2. BANF1 and lamin A/C can also bridge nucleosomes between each other. 3. In the presence of linker histone H1, H1 displaces the BANF1-lamin A/C complex from its binding sites at the nucleosomal dyad and linker DNA regions, thereby stabilizing nucleosomes. **(D)** Following physical stress, the NE ruptures, and BANF1 localizes to exposed DNA regions, recruiting a new NE through interactions with LEM domain proteins. **(E)** The DDR mechanism involving BANF1: 1. Oxidative stress induces modifications in emerin, weakening its interaction with BANF1 and promoting BANF1’s nuclear translocation. 2. BANF1 binding to PARP1 attenuates PARP1 activity. 3. BANF1 binds to and inhibits DNA-PKcs. **(F)** BANF1 competes with cGAS for DNA binding, thereby regulating the cGAS-STING pathway. TMIGD1 stabilizes BANF1 while also binding to cytoplasmic BANF1; subsequently, BANF1 captures p65, inhibits its nuclear translocation, and ultimately suppresses activation of the NF-κB pathway. **(G)** The promotional role of BANF1 in viral infection: Following retroviral infection, BANF1 acts as a component of the PIC. Gammaherpesviruses utilize BANF1 to inhibit cGAS recognition of viral DNA, attenuating the innate immune cGAS-STING pathway. ASFV also inhibits this pathway via BANF1, promoting viral replication. **(H)** BANF1 binds to vaccinia virus DNA to inhibit replication, while the virus-encoded B1 kinase phosphorylates BANF1, reversing this effect. The LEM-4-PP2A complex dephosphorylates BANF1, antagonizing B1 kinase activity.

During early mitosis, VRK1-mediated phosphorylation of BANF1 triggers its detachment from chromosomes and the NE, contributing to NE loosening and eventual disassembly, after which BANF1 passively disperses into the cytoplasm (16, 49) (**Figure 3A**). As mitosis progresses toward completion, BANF1 undergoes dephosphorylation and reassociates with chromosomes, displaying a relatively uniform distribution along their length (37, 56, 57). Notably, BANF1 colocalizes with LAP2α at telomeric regions, indicating their coordinated localization during NE reassembly and indirectly supporting their involvement in maintaining telomeric NE stability throughout interphase (58). During late anaphase and telophase, BANF1, along with the majority of LAP2α, is recruited to the central “core” region of the reforming nuclear rim at the spindle poles (56, 57, 59). BANF1 is among the earliest proteins enriched in this region, a process that is dependent on spindle microtubules (MTs) (60). Concurrently, additional constituents such as nucleoporins, lamin B, the lamin B receptor (LBR), and LAP2β are recruited to the peripheral “non-core” regions of the chromosome mass. These components play integral roles in the assembly of the nuclear pore complex (NPC), the formation of the nuclear lamina (NL), and the organization of the NE, respectively (57, 61, 62). Subsequently, the core region rapidly accumulates substantial quantities of proteins such as BANF1, lamin A/C, and emerin, culminating in the formation of a compact transmembrane chromosome bridge that facilitates NE closure (59, 60, 62) (**Figure 3B**). These observations suggest that BANF1 functions as a pioneer factor in NE reconstruction. Upon binding to chromosomes, BANF1 recruits LEM domain-containing proteins and lamin A/C to assemble functional complexes essential for NE integrity. Deficiency of BANF1 markedly impairs this reconstruction process, resulting in aberrant membrane structures (56, 60, 63).

NE remodeling encompasses complex mechanisms of membrane reorganization. The protein LEM-2 facilitates interactions between BANF1 and MTs through its LEM domain and low-complexity domain (LCD), respectively, while concurrently recruiting charged multivesicular body protein 7 (CHMP7) via its winged-helix (WH) domain. This recruitment triggers the activation of the endosomal sorting complex required for transport III (ESCRT-III) pathway, which mediates MTs disassembly and completes membrane remodeling (64, 65) (**Figure 3B**). Investigations in Caenorhabditis elegans indicate that, despite mutations in BANF1, emerin and LEM-2 can still be targeted to the NE to support its reconstruction (28). The spindle apparatus imposes spatial constraints, resulting in slower membrane remodeling in the core region than in the peripheral regions (60). Additionally, studies in Drosophila during mitotic metaphase demonstrate that a fraction of centromere-associated BANF1 (centromeric BANF1, cenBANF1) is shielded from phosphorylation by PP4, which is recruited by the constitutive centromere protein CENP-C. Concurrently, centromeric BAF inhibits PP2A activity, thereby preventing the dephosphorylation of pBANF1. This dual mechanism avoids the premature accumulation of BANF1 around chromosomes, ensuring the proper assembly of the centromere and the faithful progression of mitosis (66). In contrast, the non-phosphorylatable BANF1 mutant disrupts mitotic progression and NE dynamics by enhancing heterochromatin–NE anchoring and reducing CENP-C levels (67).

### 3.2 BANF1 in chromatin architecture and gene regulation

During late mitotic NE reassembly, BANF1 associates with chromatin DNA and facilitates the tethering of chromatin to the NE through its interaction with the Igfold domain of lamin A/C. Structural analyses have demonstrated that BANF1 dimers contribute to chromatin organization via two distinct mechanisms. First, BANF1 dimers directly bind to nucleosome linker DNA, while lamin A/C concurrently interacts with DNA at the nucleosomal dyad region, collectively stabilizing nucleosome structure. Second, the BANF1-lamin A/C complex symmetrically engages superhelical locations on nucleosomal DNA, thereby bridging adjacent nucleosomes; notably, in this mode, lamin A/C does not directly contact nucleosomal DNA (3). The presence of linker histone H1 competitively displaces the BANF1-lamin A/C complex from its binding sites on the nucleosomal dyad and linker DNA. Given lamin A/C’s involvement in both mechanisms, it is posited that BANF1 and lamin A/C cooperatively facilitate chromatin compaction and perinuclear positioning, thereby establishing the structural foundation for chromatin-NE interactions (3) (**Figure 3C**). BANF1 exhibits concentration-dependent effects: at optimal levels, it supports proper NE assembly, whereas excessive concentrations induce hypercompaction of chromatin and impede NE reformation (24). In the Drosophila nervous system, BANF1 colocalizes with the transcription factor Teashirt within heterochromatic domains, jointly mediating heterochromatin anchoring to the inner nuclear membrane. This interaction modulates transcriptional programs and preserves neural progenitor cell lineage fidelity (68). These observations suggest that BANF1 maintains nuclear architecture during the interphase of mitosis by stabilizing chromatin-NE linkages, while promoting chromatin compaction and NE remodeling during mitosis, thereby contributing to genomic stability.

BANF1 regulates gene expression and the epigenetic landscape through multiple mechanisms. It can directly interact with specific transcription factors; for example, it binds to cone-rod homeobox (CRX) to inhibit the activation of retinal genes (69). Additionally, BANF1 associates with factors such as retinoblastoma binding protein 4 (RBBP4) (70) and the promoter of the eukaryotic fusion protein gene in C. elegans (71) to regulate transcription. Indirectly, abnormal localization or dysfunction of BANF1 can activate inflammatory pathways, such as S100A9/c-Jun (72), or alter the nucleocytoplasmic distribution of transcription factors like E2F-1 (73). In embryonic stem cells, BANF1 is critical for maintaining self-renewal and pluripotency; its downregulation leads to decreased expression of key transcription factors (Sox2, Oct4, Nanog) and reduced differentiation capacity (74, 75). Moreover, BANF1 interacts with histones H1.1, H3, and H4, and also associates with chromatin regulators such as the H3-K9 methyltransferase G9a and the protein phosphatase 2A inhibitor SET/I2PP2A. These interactions enable BANF1 to regulate histone methylation, acetylation, and other post-translational modifications, thereby modulating chromatin structure and gene expression (76, 77). Additionally, BANF1 can participate in transcriptional repression and epigenetic silencing of target genes by competitively binding to emerin or by cooperating with the LEM-2/Enhancer of zeste homolog 2 complex (78, 79). Collectively, these findings position BANF1 as a pivotal regulator within gene expression and epigenetic regulatory networks.

### 3.3 BANF1 in NE rupture repair and DNA damage repair

BANF1 is integral to the repair of NE ruptures. During the interphase of mitosis, NE rupture can occur due to mechanical stress and other factors (80–83). In such events, cytoplasmic BANF1 rapidly associates with the exposed chromatin at the rupture site, serving as a scaffold to recruit LEM-domain proteins and lamin A/C, thereby initiating the repair process (34, 84–86). This recruitment is facilitated by the strong affinity of BANF1 dimers for dsDNA (59). Subsequently, LEM-2 recruits CHMP7, which activates the ESCRT-III membrane remodeling machinery to complete membrane resealing (34, 82) (**Figure 3D**). BANF1 is proposed to facilitate the rapid assembly of the “NE plaque” by establishing a localized molecular barrier, which limits nucleoplasmic leakage and safeguards genomic integrity (59, 86, 87). During this process, lamin C binds to the rupture site prior to lamin A aggregation, while BANF1 transiently aggregates before dispersing into the nucleoplasm and NE (59, 86). Similarly, during mitosis, BANF1 forms a permeability barrier around the chromosomes, also playing a protective role (88). Additionally, BANF1 competitively displaces cyclic GMP-AMP synthase (cGAS) from chromatin, thereby mitigating excessive activation of innate immune signaling during repair (89) (**Figure 3D**). Loss or downregulation of BANF1 lowers cellular tolerance to mechanical stress, increasing NE rupture frequency and delaying repair, although the overall extent of repair remains largely unaffected (34, 84). Specifically, compared to control cells, BANF1-deficient cells show a significantly delayed time to restore nuclear compartmentalization after prolonged NE rupture; however, the recovery speed after brief NE rupture is not notably affected (84). This difference may stem from the division of repair mechanisms: smaller NE ruptures can be directly repaired through bypass pathways such as nuclear envelope transmembrane proteins (NETs) or ESCRT-III, whereas BANF1 predominantly mediates the repair of larger-scale NE ruptures (59, 84, 90, 91). Further investigations have demonstrated that BANF1 is recruited in greater abundance to primary nuclear ruptures compared to micronuclei—structures associated with chromosomal breaks or instability—highlighting the critical function of the BANF1-LEM-2-CHMP7 axis in maintaining NE homeostasis (92).

BANF1 is also closely implicated in the DDR. Under conditions of oxidative stress, BANF1 modulates DDR through multiple mechanisms. Oxidative stress induces phosphorylation of the NE protein emerin and concurrently reduces its O-GlcNAcylation, modifications that weaken the interaction between emerin and BANF1. This attenuation promotes the nuclear accumulation of BANF1, thereby coordinating the cellular damage response and cell cycle regulation (93). Moreover, BANF1 directly interacts with and inhibits the core repair enzyme Poly [ADP-ribose] polymerase 1 (PARP1), limiting its ADP-ribosylation activity. Notably, mutations associated with NGPS, such as the A12T variant, enhance this inhibitory effect, exacerbating defects in DNA repair (94). BANF1 also binds to and inhibits the DNA-dependent protein kinase catalytic subunit (DNA-PKcs), thereby negatively regulating the non-homologous end-joining pathway responsible for double-strand break repair (95) (**Figure 3E**). Beyond these pathways, evidence suggests that BANF1 may functionally interact with nucleotide excision repair complexes, including the Cullin 4A-Damage-specific DNA binding protein 1-Damage-specific DNA binding protein 2 complex (70). Additionally, although Ankle1—a LEM-domain protein containing a GIY-YIG endonuclease domain—is known to play a significant role in DDR (96, 97), the potential involvement of BANF1 in modulating Ankle1 function remains to be elucidated.

### 3.4 BANF1 and innate immunity

BANF1 plays a critical role in modulating innate immune responses. Mechanistically, it suppresses the cyclic GMP-AMP synthase-stimulator of interferon genes (cGAS-STING) immune pathway by competitively binding to exposed dsDNA in the cytoplasm, thereby limiting inflammatory cytokine production (89). Upon NE rupture, genomic DNA leaks into the cytoplasm and is recognized by the DNA sensor cGAS. Cytoplasmic BANF1 displaces cGAS from DNA, reducing the synthesis of the second messenger cyclic GMP-AMP (cGAMP). This, in turn, inhibits downstream activation of STING and subsequent expression of type I interferons (IFNs) and interferon-stimulated genes (89, 98). This regulatory mechanism is particularly critical during NE rupture, as it protects cells from aberrant immune activation triggered by self-DNA (89, 98). In addition, BANF1 is implicated in the NF-κB pathway through its interaction with Transmembrane and immunoglobulin domain-containing protein 1 (TMIGD1). TMIGD1 directly binds to cytoplasmic BANF1, stabilizing the protein and concurrently suppressing inflammation by inhibiting nuclear translocation of the NF-κB subunit p65. In Crohn’s disease (CD) patients, downregulation of TMIGD1 leads to reduced BANF1 levels, resulting in diminished NF-κB inhibition and consequent exacerbation of intestinal epithelial inflammation and barrier dysfunction (99). Abnormally high BANF1 expression suppresses antitumor immune responses and promotes the development of tumor immune evasion phenotypes in various cancers, including head and neck squamous cell carcinoma (HNSCC) (13), gastric cancer (GC) (100), pancreatic ductal adenocarcinoma (PDAC) (101), T-cell desert-type triple-negative breast cancer (TCD-TNBC) (8), thyroid cancer (THCA) (102), colorectal cancer (CRC) (103), and melanoma (103) (**Table 1****;** **Figure 3F**).

**Table 1:** Summary of BANF1 functions in viral infection, cancer, and human diseases.

Disease type	Mechanism	Expression and disease role	Potential therapeutic implications	References
Crohn’s disease (CD)	TMIGD1 downregulation reduces BANF1 protein stability, which weakens BANF1-mediated suppression of p65 nuclear translocation and leads to NF-κB pathway activation.	Downregulated due to TMIGD1 loss, Promote inflammation and tissue damage	Restoring TMIGD1/BANF1 may alleviate inflammation	(99)
HIV/MMLV retroviral infection	Binds viral DNA, prevents autointegration, and facilitates viral integration	Expressed (host factor), Promotes viral replication (exploited by virus)	Inhibiting BANF1 may block viral integration	(1, 104–107)
Vaccinia virus infection	Binds viral DNA and inhibits replication; viral B1 kinase phosphorylates BANF1 to counteract its antiviral activity	Expressed (host defense factor), Antiviral (host defense)	Enhancing BANF1 activity or inhibiting B1 kinase may serve as an antiviral strategy	(22, 108–110)
HSV-1 infection	Dephosphorylated BANF1 binds viral DNA and inhibits replication; also promotes viral early gene expression (dual role)	Dephosphorylated upon infection, Dual function (antiviral vs. pro-viral)	Its dual role requires further investigation	(111, 112)
HBV infection	HBx upregulates miR-203 to suppress BANF1 expression, facilitating viral evasion of host antiviral responses	Downregulated by HBx/miR-203, Viral evasion of host defense	Restoring BANF1 expression may suppress HBV replication	(113)
Hepatocellular carcinoma (LIHC)	Promotes cell cycle progression and EMT; serves as an early diagnostic marker	High, Pro-tumorigenic; early diagnostic marker	Targeting BANF1 may serve as an early intervention strategy	(12, 114, 115)
Colorectal cancer (CRC)	Promotes EMT, proliferation, and migration; upregulates GLI1/Cyclins/BCL2; suppresses the cGAS-STING pathway to mediate immune evasion	High, Pro-tumorigenic; indicates poor prognosis	BANF1 knockdown may enhance the efficacy of PD-1 blockade	(10, 103, 116)
Esophageal squamous cell carcinoma (ESCC)	VRK1 phosphorylates BANF1 to promote proliferation	High, Pro-tumorigenic	The VRK1-BANF1 axis may serve as a therapeutic target	(117)
Glioblastoma (GBM)	VRK2 methylation silencing renders VRK1 a synthetic lethal target; loss of BANF1 function leads to nuclear abnormalities and G2/M arrest	High, Pro-tumorigenic	VRK1 inhibitors (e.g., luteolin) are effective in VRK2-deficient GBM	(118, 119)
Triple-negative breast cancer (TNBC)	BANF1 loss leads to loss of NE integrity, mitotic arrest, and cell death; suppresses immunity	High, Pro-tumorigenic	Potential prognostic marker and therapeutic target	(8, 120)
Pancreatic ductal adenocarcinoma (PDAC)	Member of an HOXA10-driven 5-gene signature; mediates immune suppression	High, Pro-tumorigenic	Potential prognostic marker and therapeutic target	(101, 121)
Gastric cancer (GC)	Shapes the TME; negatively correlates with immune infiltration	High, Pro-tumorigenic; poor prognosis; influences immunotherapy response	Prognostic marker and potential immunotherapy target	(100)
Head and neck squamous cell carcinoma (HNSCC)	Associated with cell adhesion pathway; negatively correlates with immune infiltration	High, Pro-tumorigenic; poor prognosis; influences immunotherapy response	Prognostic marker and potential therapeutic target	(13)
Lung adenocarcinoma (LUAD)	Contributes to tumor immune evasion; decreased ESTIMATE score; promotes EMT	High, Contributes to tumor immune evasion; decreased ESTIMATE score; promotes EMT	Immune and prognostic marker; knockdown suppresses tumor proliferation	(122)
Thyroid carcinoma (THCA)	BANF1 suppresses CD8^+^ T cell proliferation via the PI3K/AKT/mTOR pathway	High, Pro-tumorigenic; affects immune infiltration	Targeting BANF1 may modulate CD8^+^ T cell activity via the PI3K/AKT/mTOR pathway, thereby enhancing anti-tumor immunity	(102)
Cervical cancer (CC)	The IRF1/miR-203 axis downregulates BANF1; BANF1 interacts with p16 and sequesters it in the cytoplasm	High, Tumor-suppressive (BANF1 is downregulated); p16 function is antagonized	Restoring miR-203 or targeting the BANF1-p16 interaction may be effective	(123, 124)
Néstor-Guillermo progeria syndrome (NGPS)	Mutation reduces lamin A/C affinity; defective NE repair; mitotic abnormalities	Compensatory elevation, Pathogenic (loss or alteration of function)	Targeting PARP1 or restoring NE repair	(15, 85, 125, 126)
Dominant Motor Neuronopathy	Mutation enhances dsDNA affinity; mitotic abnormalities	No significant change, Pathogenic (gain or alteration of function)	Pathogenic (gain or alteration of function)	(14)
Alzheimer’s disease (AD)	BANF1 promotes ROS production and glutamate-induced apoptosis in HT22 cells	Pathological elevation, Promotes neurodegeneration	Targeting BANF1 may slow neuronal loss	(18)

### 3.5 BANF1 in antiviral defense and viral resistance

BANF1 exerts context-dependent, dual functions in viral infections, acting either as a proviral host factor or as a restrictive antiviral component. In the context of antiviral defense, BANF1 exhibits dichotomous functions. On one hand, it serves as a component of the pre-integration complex (PIC) in retroviruses such as human immunodeficiency virus (HIV) and MMLV, where it compacts viral DNA to prevent autointegration and thereby promotes efficient proviral integration (1, 104–107). This function is modulated by BANF1 phosphorylation, which disrupts its incorporation into the PIC (46). Conversely, several viruses exploit BANF1 to evade innate immune surveillance. For instance, gammaherpesviruses utilize BANF1 to suppress cGAS-mediated recognition of viral DNA, thereby attenuating the cGAS-STING pathway (127). Similarly, although no interaction between BANF1 and viral DNA has been found in African swine fever virus (ASFV), knocking down BANF1 significantly enhances the cGAS-STING pathway’s inhibition of viral replication (128). BANF1 and its phosphorylated forms have also been shown to promote early gene transcription and replication of white spot syndrome virus (129). In addition, BANF1 expression is upregulated in condyloma acuminatum lesions caused by human papillomavirus (HPV) infection, although the functional significance of this observation remains to be elucidated (130) (**Table 1****;** **Figure 3G**).

Conversely, BANF1 also exerts direct antiviral effects through its DNA-bridging activity (108, 109). For vaccinia virus, which replicates in the cytoplasm, BANF1 can bind to the viral DNA and inhibit its replication; however, the viral B1 kinase counteracts this inhibition by phosphorylating BANF1, while the host PP2A can dephosphorylate pBANF1 to restore its activity (22, 108–110). Recent studies have found that LEM-4 also participates in this dephosphorylation process through interaction with PP2A (**Figure 3H**), and the LEM-4 mutant lacking the transmembrane domain exhibits an even stronger promoting effect (131). The role of BANF1 in herpes simplex virus 1 (HSV-1) infection is species-specific, as HSV-1 replicates within the nucleus. In mice, BANF1 undergoes dephosphorylation, which facilitates its translocation into the nucleus, where it inhibits gene expression by binding to viral DNA; non-phosphorylated mutants demonstrate even greater inhibitory efficiency (111). In contrast, in common human cell lines, BANF1 promotes early viral gene expression and enhances viral replication by recruiting the SETD1A methyltransferase to the viral promoter region (112). This discrepancy may be attributed to species differences, cell type variations, or differences in experimental conditions; however, further investigation is required to clarify these mechanisms. Additionally, BANF1 overexpression suppresses hepatitis B virus (HBV) replication; this effect is antagonized by the viral accessory protein HBx, which upregulates miR-203 and thereby downregulates BANF1 expression (113). Collectively, these findings illustrate that BANF1 operates as a dynamic and context-dependent regulator within the virus-host interface, exerting both proviral and antiviral functions depending on the specific pathogen and cellular environment (**Table 1**).

## 4 Analysis of BANF1 expression in different tumors

**Figure 4:**
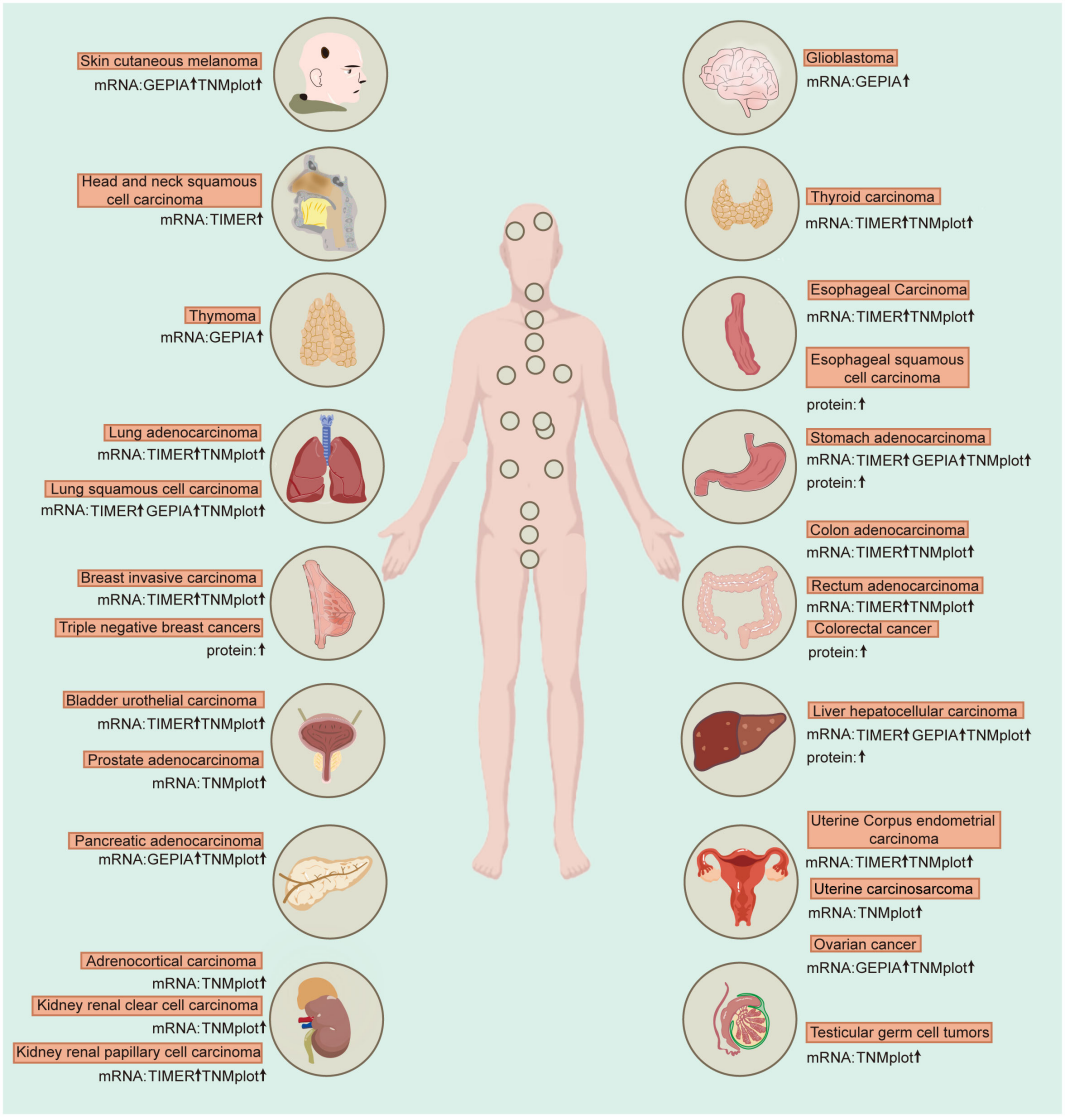
Expression levels of BANF1 in different tumors. Analysis of the TIMER, GEPIA, and TNMplot databases reveals consistent upregulation of BANF1 mRNA across a broad range of malignancies, including skin cutaneous melanoma, HNSCC, thymoma, lung adenocarcinoma, lung squamous cell carcinoma, breast invasive carcinoma, bladder urothelial carcinoma, prostate adenocarcinoma, pancreatic adenocarcinoma, adrenocortical carcinoma, kidney renal clear cell carcinoma, kidney renal papillary cell carcinoma, glioblastoma, THCA, esophageal carcinoma, ESCC, stomach adenocarcinoma, colon adenocarcinoma, rectum adenocarcinoma, LIHC, uterine corpus endometrial carcinoma, uterine carcinosarcoma, ovarian cancer, and testicular germ cell tumors. Elevated BANF1 protein levels have been further confirmed by immunohistochemistry in ESCC, LIHC, TNBC, CRC, and stomach adenocarcinoma.

Recent studies have found that BANF1 is abnormally expressed in various cancers. The expression levels of BANF1 were analyzed across 31 distinct tumor types using the GEPIA, TIMER, and TNMplot databases (132–134). The analysis revealed that BANF1 mRNA expression was significantly elevated in 26 tumor types compared to their corresponding normal tissues. At the protein level, immunohistochemistry confirmed that BANF1 expression was higher in CRC (10), GC (11), TNBC (135), ESCC (9), and LIHC (12) compared to normal tissues (**Figure 4**). Although factors such as geographic origin, sample size, detection methods, and tumor heterogeneity may cause discrepancies among different studies, these data collectively provide an important foundation for exploring the expression and functional significance of BANF1 in human malignancies.

Clinically, high BANF1 expression is associated with lymph node metastasis in GC, TNBC, and HNSCC (11, 13, 135). Prognostic analyses indicate that elevated BANF1 expression correlates with shorter overall survival and disease-free survival in patients with CRC, GC, and HNSCC (10, 11, 13, 100, 136). In addition, BANF1 expression exhibits a dual role in treatment response: in HNSCC, tumors with high BANF1 expression are more sensitive to immune checkpoint inhibitors (13), whereas in TNBC, knocking down BANF1 can enhance chemotherapy sensitivity to doxorubicin (137). These observations suggest that BANF1 may serve not only as a potential prognostic biomarker but also influence treatment outcomes across various cancer types.

### 4.1 The mechanism of BANF1 in tumorigenesis and tumor progression

BANF1 is commonly overexpressed in various tumors, and its expression level is closely associated with tumor progression, patient prognosis, and treatment sensitivity. Functional studies have preliminarily elucidated several mechanisms by which BANF1 promotes tumor development and progression. Although there are differences depending on the tumor type, a relatively core set of common functions has emerged.

#### 4.1.1 Cell cycle and mitosis regulation

**Figure 5:**
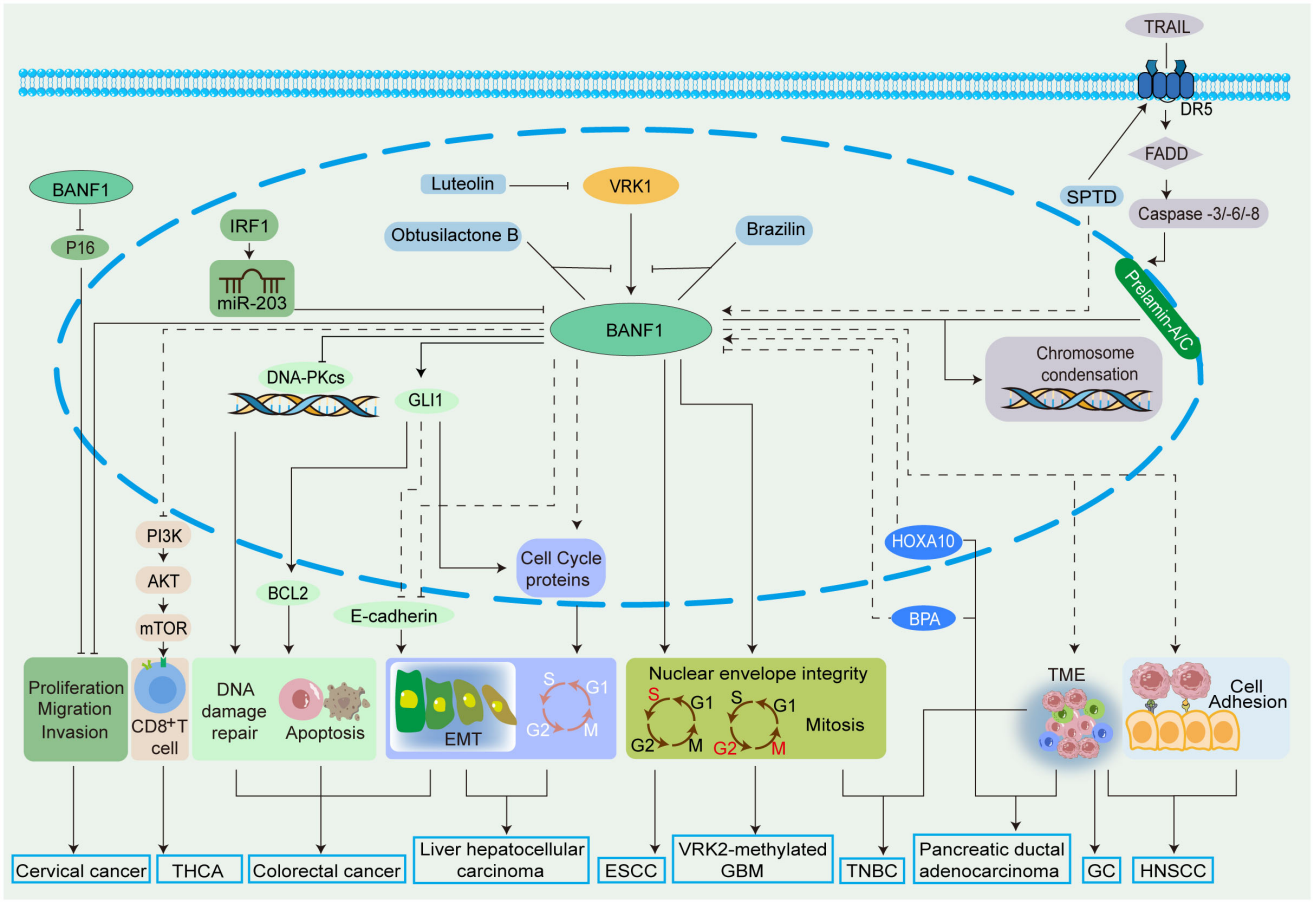
BANF1 mechanisms in tumors and targeted drugs. (1) Cell cycle and mitosis: In LIHC, BANF1 inactivation suppresses tumorigenesis by selectively modulating EMT and cell cycle proteins. In CRC, the VRK1/BANF1/GLI1 axis upregulates cyclins and BCL2 to drive proliferation and survival. In ESCC, GBM, and TNBC, VRK1 depletion dampens BANF1 activity, promoting cell cycle arrest and nuclear abnormalities. (2) NE instability and DDR: In CRC, BANF1 modulates the DDR by inhibiting repair proteins such as DNA-PKcs. In GBM and TNBC, BANF1 suppression leads to nuclear aberrations and restricts tumor growth. (3) EMT and metastasis: BANF1 overexpression is accompanied by classical EMT marker changes, most notably E-cadherin loss. This manifests through the VRK1/BANF1/GLI1 axis in CRC and through cell adhesion pathway dysregulation in HNSCC. (4) TME and immune evasion: High BANF1 expression correlates negatively with immune infiltration in HNSCC, GC, PDAC, and TNBC. In THCA, BANF1 suppresses CD8^+^ T cell proliferation via the PI3K/AKT/mTOR pathway. (5) Cancer type-specific mechanisms: In PDAC, BANF1 serves as an HOXA10-driven prognostic gene and is associated with BPA exposure. In CC, the IRF1/miR-203 axis represses BANF1 expression, and BANF1 can sequester p16 in the cytoplasm, counteracting its tumor-suppressive function. (6) Therapeutic strategies: Obtusilactone B and brazilin bind BANF1 directly and interfere with VRK1-mediated phosphorylation. Luteolin targets VRK1. SPTD upregulates BANF1 while engaging the DR5/FADD/caspase cascade to promote prelamin-A/C–BANF1 interaction and drive chromatin condensation.

BANF1 fuels tumor cell proliferation through its control of cell cycle progression. In LIHC, coordinated inactivation of BANF1, procollagen-lysine, 2-oxoglutarate 5-dioxygenase 3 (PLOD3), and splicing factor B subunit 4 (SF3B4) suppresses tumorigenesis by selectively modulating EMT and cell cycle proteins (12, 114, 115). In CRC, BANF1 upregulates glioma-associated oncogene homolog 1 (GLI1), which in turn drives the expression of cell cycle facilitators such as Cyclin D1 and anti-apoptotic factors such as B-cell lymphoma/leukemia-2 (BCL2), thereby enhancing both the proliferative capacity and survival of tumor cells (10, 116). VRK1, a nuclear kinase, further contributes to this circuit by phosphorylating BANF1 to regulate mitotic progression; its depletion traps BANF1 on chromatin and causes defective NE rupture (44, 138). In CRC, VRK1 and BANF1 are positively correlated, and VRK1 knockdown lowers the levels of both BANF1 and GLI1, restraining tumor development (10). In ESCC, loss of VRK1 similarly downregulates BANF1 and arrests cells in S phase, hinting that VRK1 influences proliferation, at least in part, through BANF1 (117) (**Table 1****;** **Figure 5**).

Notably, in glioblastoma (GBM) harboring VRK2 promoter methylation, VRK1 depletion likewise dampens BANF1 activity, producing nuclear morphological defects, G2-M arrest, and growth suppression (118, 119). That VRK1 loss triggers distinct cell cycle arrest phenotypes in different cancer types is an observation that merits deeper exploration. In TNBC, BANF1 loss also culminates in mitotic arrest and cell death (120). Together, these findings place BANF1 at the center of cell cycle control in cancer cells (**Table 1****;** **Figure 5**).

#### 4.1.2 NE instability and DNA damage repair

BANF1 safeguards genome stability through its dual roles in NE integrity and the DDR. Under oxidative stress, BANF1 modulates the DNA damage response by directly binding and restraining key repair proteins such as PARP1 and DNA-PKcs (93–95). This function is particularly relevant in the cancer setting, where active proliferation is frequently accompanied by NE rupture and attendant DNA damage (139, 140). In CRC, BANF1 depletion downregulates DNA-PKcs, KU70, KU80, and RAD51, thereby heightening radiosensitivity (10). In both TNBC (120) and GBM (118, 119), loss or dampening of BANF1 activity compromises NE integrity, leading to nuclear aberrations and mitotic arrest that ultimately curtail tumor growth. These observations point to a model in which elevated BANF1 expression helps cancer cells preserve both NE architecture and genome integrity (**Table 1****;** **Figure 5**).

#### 4.1.3 Epithelial-mesenchymal transition and regulation related to cell adhesion

BANF1 promotes the migration, invasion and metastasis of tumor cells by regulating the EMT process. In CRC, overexpression of BANF1 is associated with down-regulation of E-cadherin and up-regulation of N-cadherin and vimentin, which are classic EMT markers (10, 116). The VRK1/BANF1/GLI1 axis promotes CRC metastasis by enhancing proliferation, inhibiting apoptosis and regulating EMT (10, 141). In LIHC, up-regulation of E-cadherin and down-regulation of N-cadherin were also observed after targeted knockout of BANF1 (12, 114). In Lung adenocarcinoma (LUAD), it was also found that BANF1 knockdown led to upregulation of E-cadherin and downregulation of N-cadherin, promoting EMT (122). In addition, it was found in HNSCC that the differential genes related to BANF1 were mostly enriched in the cell adhesion pathway (13), suggesting that the specific mechanism by which BANF1 promotes the invasion of HNSCC may be related to EMT (**Table 1****;** **Figure 5**).

#### 4.1.4 Tumor microenvironment and immune regulation

BANF1 reshapes the TME by influencing immune signal transduction. Cytoplasmic BANF1 competes with cGAS for binding to dsDNA, thereby restricting the activation of the CGAS-STING pathway and reducing the production of immune factors such as type I IFNs (89, 98). In cancer, this mechanism may cause tumor cells to evade immune surveillance after NE rupture. Conversely, BANF1 depletion can reactivate cGAS-STING signaling and enhance anti-tumor immunity (103). In CD, the down-regulation of TMIGD1 reduces the level of BANF1, relives the inhibition of NF-κB and promotes intestinal inflammation, increasing the risk of CRC (99, 142). It was also found that the expression of BANF1 was negatively correlated with the immune response in HNSCC (13), GC (100), PDAC (101), and TCD-TNBC (8). Specifically, knockdown of BANF1 was found in CRC and melanoma to enhance the immune efficacy of programmed cell death protein-1 (PD-1) blockade (103). Furthermore, in THCA, BANF1 acts on CD8^+^ T cells through the phosphatidylinositol 3-kinase/protein kinase B/mammalian target of rapamycin (PI3K/AKT/mTOR) pathway. Knockdown of BANF1 can promote the proliferation of CD8^+^ T cells and enhance immune infiltration (102) (**Figure 5**). However, the relationship between BANF1 and CD8^+^ T cells is not completely consistent. Meihan Liu et al. (122) found that BANF1 was positively correlated with CD8^+^ T cells in LUAD, while the ESTIMATE score showed that BANF1 was negatively correlated with the immune score. It is believed that CD8^+^ T cells in BANF1 high tumors may be enriched due to functional limitations rather than functional anti-tumor phenotypes, but this hypothesis still requires further experimental verification (**Table 1**).

#### 4.1.5 Other mechanisms

In addition to the above-mentioned common functions, BANF1 also exhibits unique regulatory patterns in some cancers. In PDAC, BANF1 is both A regulatory gene for Bisphenol A (BPA) exposure interfering with pancreatic function and a characteristic member of HOXA10-driven prognosis-related 5 genes. Its high expression is associated with short survival in patients and may promote disease progression by mediating tumor immunosuppression (101, 121). In cervical cancer (CC), IRF1 drives the transcription of miR-203, which targets and binds to the 3’UTR of BANF1 and down-regulates its expression. The reduction of BANF1 can inhibit the clone formation, migration and invasion abilities of CC cells (123). In addition, BANF1 can interact with cytoplasmic p16, trapping it in the cytoplasm and hindering its nuclear anti-tumor function. This results in overexpression of p16 in CC still failing to inhibit tumor growth and is associated with a poor prognosis (124) (**Table 1****;** **Figure 5**).

### 4.2 Therapeutic strategies targeting BANF1

The therapeutic strategy targeting BANF1 exhibits the characteristics of multi-target and multi-pathway coordinated intervention, with broad prospects for drug development and strong potential for clinical transformation. At present, the therapeutic strategies targeting BANF1 are mainly based on the regulatory effect of its phosphorylation modification on nuclear membrane homeostasis. On the one hand, obtusilactone B and brazilin can directly bind to BANF1, interfering with VRK1-mediated phosphorylation modification, causing them to abnormally maintain the connection between the nuclear membrane and DNA, thereby inducing mitotic arrest and inhibiting tumor growth (143–146). Computer simulation analysis shows that the oxidation product of brazilin, brazilein, has higher stability when combined with BANF1 (144). Rabeprazole can bind BANF1 under oxidizing conditions and disrupt its interaction with LEM-domain proteins, thereby facilitating the nuclear delivery of exogenous DNA; however, its inhibitory efficiency has not been recapitulated *in vivo* (21, 147). On the other hand, targeting the upstream kinase VRK1 can indirectly regulate BANF1 function. For instance, Luteolin inhibits VRK1 kinase activity by targeting and reducing the phosphorylation level of BANF1, inducing cell cycle arrest and weakening the proliferation and survival ability of tumor cells (148) (**Table 2****;** **Figure 5**). It is worth noting that although the SpyTFAMoplex complex designed by taking advantage of the phosphorylation of BANF1 by VRK1 was intended to antagonize BANF1 capture and promote exogenous DNA transfection, studies have found that the transfection efficiency of kinase-inactivated VRK1 is actually higher. Hint at the complexity of regulating the BANF1 function in the design of non-viral vectors (150, 151).

**Table 2:** Summary of investigational agents targeting BANF1 and its signaling axis.

Intervention	Molecular target	Mode of action	Signaling pathways	Disease type	References
Obtusilactone B	BANF1 (direct binding)	Directly binds BANF1, interferes with VRK1-mediated phosphorylation, disrupts its normal interaction with DNA and the NE, and induces mitotic arrest	BANF1-DNA/NE interaction; BANF1 phosphorylation	Tumor (pan-cancer)	(143, 146)
Brazilin	BANF1 (direct binding)	Directly binds BANF1, interferes with VRK1-mediated phosphorylation, disrupts its normal interaction with DNA and the NE, and induces mitotic arrest	BANF1-DNA/NE interaction; BANF1 phosphorylation	Tumor (pan-cancer)	(144, 145)
Rabeprazole	BANF1 (direct binding)	Binds BANF1, inhibits its interaction with LEM-domain proteins, and facilitates nuclear delivery of exogenous DNA	BANF1-LEM interaction	Non-viral gene therapy (enhances gene delivery)	(21, 147)
Luteolin	VRK1 (kinase inhibitor)	Inhibits VRK1 kinase activity, reduces BANF1 phosphorylation, and induces cell cycle arrest	VRK1-BANF1 axis	Tumor (pan-cancer)	(148)
Strophanthidin (SPTD)	DR5 (TRAIL-R2); BANF1	Activates the DR5-caspase cascade and induces nuclear chromatin condensation and apoptosis via prelamin-A/C–BANF1 signaling	DR5/caspase axis; prelamin-A/C/BANF1 interaction	LUAD	(149)
BANF1-targeted gene knockout	BANF1 (genetic level)	BANF1 knockout activates the cGAS-STING pathway, enhances anti-tumor immunity	cGAS-STING pathway	Melanoma, colon cancer; enhances PD-1 blockade efficacy	(103)

In addition, BANF1 is also involved in the synergistic enhancement of targeted signaling pathways and immunotherapy. For instance, the cardioside drug strophanthidin (SPTD) on the one hand upregulates the expression of BANF1 On the other hand, the Fas-associated protein with death domain (FADD) can be recruited through the tumor necrosis factor -related apoptosis-inducing ligand receptor 2 (TRAIL-R2, DR5) receptor to activate the caspase cascade reaction, promote the expression of prelamin-A/C, and interact with BANF1 Induce chromatin condensation in the nucleus and induce apoptosis of LUAD cells (149) (**Figure 5**). More importantly, directly inhibiting endogenous BANF1 in tumor cells can activate the cGAS-STING pathway, thereby trigger the body’s anti-tumor immune response and significantly enhance the efficacy of PD-1 blockade. It provides a new potential strategy for combined immunotherapy (103) (**Table 2**). For instance, in the MC38 colon cancer and B16F10 melanoma models, BANF1 knockout significantly inhibited tumor growth in immune-normal mice but was almost ineffective in immune-deficient nude mice, demonstrating the immune-dependent anti-tumor mechanism of BANF1-targeted therapy (103). The combination of BANF1 knockout and anti-PD-1 not only outperforms either monotherapy, but also induces persistent anti-tumor immune memory, and the cured mice can reject the re-inoculation of wild-type tumor cells (103). These results suggest that the expression of BANF1 may be related to the tumor immunosuppressive microenvironment and the poor prognosis of immunotherapy. These findings collectively suggest that targeting BANF1 not only achieves anti-tumor effects through direct intervention of its function but also can be combined with immunotherapy, presenting significant potential for clinical translation.

## 5 Relevant roles of BANF1 in medical diseases

### 5.1 Association between BANF1 and Néstor-Guillermo progeria syndrome

**Figure 6:**
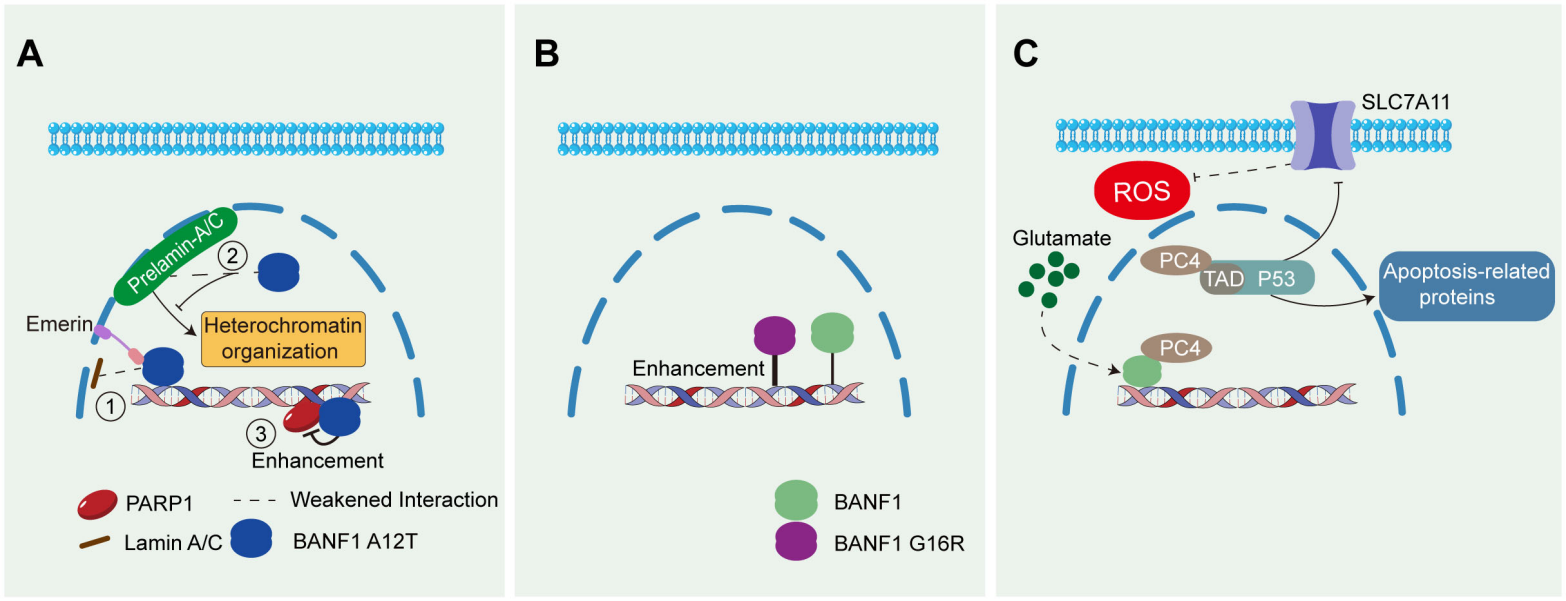
BANF1 and other human diseases. **(A)** Molecular mechanisms through which BANF1 A12T contributes to NGPS. 1. A12T impairs binding to lamin A/C. 2. A12T disrupts the interaction with prelamin-A/C. 3. A12T enhances binding to PARP1, thereby suppressing its activity. **(B)** The BANF1 G16R mutation enhances binding to dsDNA. **(C)** Glutamate induces BANF1 expression; BANF1 binds PC4, which in turn engages the TAD of p53 to modulate downstream effects.

Néstor-Guillermo progeria (NGPS) is a rare autosomal recessive genetic disorder caused by a homozygous missense mutation (A12T) in the BANF1 gene (15). Its clinical features include typical progeria manifestations such as alopecia and osteoporosis. However, compared with Hutchinson-Gilford progeria syndrome, patients with NGPS usually have normal cognitive function, longer survival period, and less severe cardiovascular and metabolic complications (15, 152, 153). At the molecular mechanism level, it is generally believed that the A12T mutation specifically reduces the affinity for lamin A/C (85, 125, 126). However, it does not affect dimerization, protein structure, and their binding ability to DNA and LEM-domain proteins (15, 16, 125). Another study pointed out that this mutation reduced the binding ability to DNA (23, 154), but its ability to recruit DNA from the NE rupture site was not significantly affected (125, 155). Due to the proximity of the mutation location to the lamin A/C binding region (23), this reduces the recruitment efficiency of BANF1 for lamin A/C during the NE rupture repair process, thereby affecting the repair speed of NE and the structural stability after repair (85, 155). To compensate for this functional defect, the total protein level of BANF1 in NGPS cells increased compensatorily (85). Research in the Drosophila progeria mutation model has found that this mutation can interfere with the mitotic process of germ stem cells and somatic cells, specifically manifested as abnormal spindle and centromeric functions (156). This mitotic defect can activate Checkpoint kinase 2 in the DDR pathway and selectively induce apoptosis in cells with differentiation defects or carrying mutations. This process differs from the cell death pattern caused by complete deletion of BANF1 (156, 157). Furthermore, the A12T mutation also weakened the interaction between BANF1 and prelamin-A/C, possibly affecting heterochromatin organization (158, 159); It even enhances the binding with PARP1, further inhibiting its activity (94). In conclusion, the homozygous mutation of BANF1 (A12T) leads to progeria phenotype by influencing multiple cellular regulatory mechanisms. However, the differences in its effects in different tissue cells and the specific regulatory details of the DNA repair pathway still need to be further explored (**Table 1****;** **Figure 6A**).

### 5.2 Association between BANF1 and dominant motor neuronopathy

In 2023, Agathe Marcelot et al. (14) first reported a dominant motor neuropathy caused by a *de novo* mutation G16R in the BANF1 gene. The main clinical manifestation is progressive myasthenia, which has a progressive impact on the patient’s motor function. Moreover, neither of the patient’s parents carries this mutation. This discovery enriches the pathogenic gene spectrum of motor neuropathy. Unlike the A12T mutation, the G16R mutation did not cause significant structural abnormalities in the nuclear lamina. The pathogenic mechanism lies in that this mutation changes the protein conformational balance by introducing a salt bridge, abnormally enhancing the affinity of BANF1 for dsDNA and causing it to remain on chromatin (**Table 1****;** **Figure 6B**). This “function-acquired” abnormal combination may lead to disorders in local chromatin states and gene expression programs, specifically affecting the homeostasis and function of motor neurons, but its specific downstream pathways still need to be clarified.

### 5.3 Association between BANF1 and Alzheimer’s disease

Alzheimer’s disease (AD) is a common neurodegenerative disorder in the elderly population. Its specific mechanisms are related to genetic factors, Amyloid-β aggregation, Tau hyperphosphorylation and glutamate toxicity, etc. (18, 160). Studies have found that excessive glutamic acid leads to an increase in the expression level of BANF1 in mice, promotes the production of reactive oxygen species (ROS), and thereby induces apoptosis (18). It is known that BANF1 can interact with the transcriptional cofactor PC4 (70). PC4 can bind to the N-terminal transactivation domain (TAD) of P53, activate p53, thereby up-regulating the expression of related apoptotic proteins and inhibiting the expression of the System Xc SLC7A11 subunit. This led to an increase in ROS levels (18) (**Table 1****;** **Figure 6C**). Therefore, it is speculated that BANF1 may play a certain role in the pathological process of AD by interfering with the functions of complexes such as PC4-p53 and participating in the regulation of oxidative stress levels and apoptotic signals within neurons. However, its exact mechanism and causal association still need further research for confirmation (18).

Based on the above analysis, the pathogenic mechanism of BANF1 in human diseases can be summarized into three patterns, which form a sharp contrast with the situation in tumors (1). Loss-of-function type (NGPS): The homozygous missense mutation of BANF1 (A12T) weakens the protein function, reduces its recruitment efficiency for lamin A/C, and leads to a slower repair speed of nuclear membrane rupture and a decrease in structural stability after repair (85, 155) (2); Pure functional acquisition type (dominant motor neuropathy): The BANF1 heterozygous missense mutation (G16R) abnormally enhances the DNA binding affinity of proteins, causing disease through chromatin modification disorders (14) (3); Environmental-driven type (AD): Pathological stimulation (glutamate toxicity) secondary induces the upregulation of BANF1 wild-type protein expression, which participates in neuronal oxidative damage as a downstream effector molecule (18). Unlike the above three patterns, functional mutations in the coding region of BANF1 rarely occur in tumors. Database analyses such as TCGA show that the main pathogenic mechanism of BANF1 in malignant tumors is the upregulation of wild-type protein expression (10, 12, 13, 100, 102, 120, 122). In CRC, PDAC and CC, the high expression of BANF1 is mostly mediated by the abnormal expression of its upstream regulatory factors respectively, including VRK1 (117), HOXA10 (101), and miR-203 (123); In addition, in PDAC, environmental factor BPA exposure was also observed to drive an increase in BANF1 expression (121). This dichotomy of “tumor reexpression and genetic lesion mutation” reveals the core mechanism differences: Tumors gain selective advantages by dose enhancement of the inherent functions of BANF1 (such as NE protection, immune escape and proliferation promotion, etc.) (10, 12, 13, 100, 102, 120, 122), while hereditary BANF1 diseases cause cellular homeostasis collapse due to qualitative changes in specific protein functions (14, 85). This insight carries important implications for precision therapeutic strategies: tumors may require inhibition of BANF1 function, NGPS may benefit from functional enhancement or correction, and dominant motor neuronopathy may demand blockade of the mutant protein’s aberrant gain of function.

## 6 Research perspectives and challenges

As a multifunctional molecule involved in maintaining NE integrity, regulating chromatin organization, and participating in DDR and innate immunity, BANF1 has become an important target in disease mechanism research. However, existing studies have mostly focused on expression profile analysis and phenotype associations, with a lack of in-depth exploration of its precise molecular mechanisms. This study focuses on three core scientific questions: (1) Whether BANF1 is simply a structural protein or a regulatory hub integrating multiple signaling pathways requires further clarification and explanation. Current research indicates that BANF1 is not only a “building block” for maintaining NE structure but also a signaling integration node connecting NE integrity, chromatin organization, DDR, innate immunity, and disease progression. Through a complex network of protein-protein interactions, BANF1 translates physical structural changes into cellular signaling events. During NE rupture, it participates both in physical repair and, by competitively binding DNA, limits the overactivation of the cGAS-STING pathway, exemplifying the unification of structural and signaling functions. (2) Whether the role of BANF1 in different diseases is context-dependent remains to be further validated. BANF1 is generally highly expressed in tumors and promotes malignant progression; in NGPS, mutations lead to loss of function, causing premature aging; and in dominant motor neuronopathy, mutations cause gain of function, triggering neurodegeneration. This context dependency suggests that therapeutic strategies targeting BANF1 need to consider the specific disease background and cell type. In tumors, it may be necessary to inhibit BANF1 function, whereas in NGPS, enhancing its function might be required. (3) It is still unclear whether BANF1 is a causal driver of disease or merely a concomitant marker. In NGPS and dominant motor neuronopathy, BANF1 mutations are confirmed pathogenic causes, establishing its role as a causal driver. In tumors, high BANF1 expression is associated with poor prognosis, and functional studies have confirmed its role in promoting proliferation, migration, and drug resistance, supporting its driver role. However, in some tumors, abnormal BANF1 expression may be a byproduct of genomic instability or other oncogenic events. Clarifying these key issues will provide a theoretical foundation for precise diagnostic and therapeutic strategies targeting BANF1.

Current research on mechanisms faces four major bottlenecks: a lack of tissue-specific animal models to simulate physiological functions; insufficient *in vivo* causal validation, with conclusions mostly limited to the cellular level; unclear dynamic assembly mechanisms of the BANF1 protein complex; and a broken chain of translational evidence from basic discoveries to clinical applications. In terms of drug development, BANF1 is a small molecule with a compact structure, making direct targeting and draggability limited. Moreover, since it is widely involved in fundamental life processes, systemic intervention may pose a high risk of toxicity. Identifying a precise “therapeutic window” remains a core challenge.

Given the core function of BANF1 in mitosis and genomic maintenance, the potential adverse reactions of its systemic inhibition on normal proliferating tissues such as bone marrow hematopoiesis, intestinal epithelium and germ cells deserve special attention. Knockdown of BANF1 impactions the self-renewal and survival of embryonic stem cells. In germ cells, impaired BANF1 function can lead to mitotic defects, chromosomal segregation abnormalities and checkpoint kinase 2-mediated germ cell death (74, 156, 157); In the intestinal epithelium, the down-regulation of BANF1 expression is associated with barrier dysfunction and increased inflammation (99). In addition, BANF1 deficiency may also lead to genomic instability and chronic inflammation mediated by the cGAS-STING pathway (98). Therefore, in the future, therapeutic strategies targeting BANF1 should give priority to tumor-specific delivery or conditional knockout strategies to minimize off-target toxicity to normal proliferating tissues.

Despite the severe challenges, there are still several strategies that hold promise for achieving therapeutic Windows. Firstly, taking advantage of the differential dependence of tumor cells on BANF1 compared to normal cells, BANF1 overexpression exists in many malignant tumors. Tumor cells with inherent genomic instability may be more dependent on BANF1-mediated DNA repair and nuclear membrane integrity maintenance for survival. Secondly, indirectly targeting the upstream and downstream regulatory axes of BANF1 can avoid the structural draggability limitations of direct targeting of BANF1 and enhance tumor selectivity. For instance, targeting the upstream kinase VRK1 can indirectly regulate the function of BANF1 (148). In addition, the optimization of tumor-specific delivery systems and administration regimens can further reduce systemic drug exposure. Methods such as nanoparticle carriers (161), antibody-drug conjugations (162), or intratumoral local administration (163) can prioritize the enrichment of therapeutic drugs in tumor tissues, minimize drug exposure in highly proliferating normal organs, and further expand the range of safe treatment.

Future research should focus on three main directions: (1) deeply elucidating the specific regulatory mechanisms of BANF1 in tumors, NGPS, and neurodegenerative diseases to establish a theoretical foundation for targeted interventions; (2) systematically investigating its immunoregulatory role within the TME to reveal how it reshapes microenvironment composition and signal transduction; (3) advancing translational medicine research by integrating organoid models, humanized models, and clinical trials to accurately assess its efficacy and safety. With the rapid development of precision medicine and multi-omics technologies, BANF1 is expected to become a key breakthrough linking basic mechanisms to clinical therapies.

## 7 Conclusions

In summary, this review systematically outlines the basic structure and core functions of BANF1, summarizes its expression patterns, functional characteristics, and close associations with the invasive and metastatic capabilities of various tumor cells, immune evasion mechanisms, and patient clinical outcomes, and clarifies its key role in maintaining NE homeostasis, DDR, and regulation of innate immune pathways. Additionally, this article highlights the significant value of BANF1 in non-tumor diseases, including NGPS, AD, and dominant motor neuronopathy. Furthermore, the review systematically evaluates the feasibility of BANF1 as a molecular biomarker for disease diagnosis and as a potential therapeutic target, providing new research ideas and directions for precise diagnosis and treatment of related diseases. Looking ahead, with ongoing advances in mechanistic studies, specific regulators targeting BANF1 are expected to be successfully developed and applied clinically, opening new avenues for the treatment of BANF1-related diseases.

## Glossary

A12T: Ala12Thr
AD: Alzheimer’s disease
ASFV: African swine fever virus
BAF-L: BAF-like
BANF1: Barrier-to-Autointegration Factor 1
BCL2: B-cell lymphoma/leukemia-2
BPA: Bisphenol A
CC: Cervical cancer
CD: Crohn’s disease
cenBANF1: Centromeric BANF1
CENP: Centromere protein
cGAMP: Cyclic GMP-AMP
cGAS: Cyclic GMP-AMP synthase
CHMP7: Charged multivesicular body protein 7
CRC: Colorectal cancer
CRX: Cone-rod homeobox
DDR: DNA damage repair
DNA-PK: DNA-dependent protein kinase
dsDNA: Double-stranded DNA
EMT: Epithelial-mesenchymal transition
ESCC: Esophageal squamous cell carcinoma
ESCRT-III: Endosomal sorting complex required for transport III
FADD: Fas-associated protein with death domain
G16R: Gly16Arg
GBM: Glioblastoma
GC: Gastric cancer
GLI1: Glioma-associated oncogene homolog 1
HBV: Hepatitis B virus
HhH: Helix-hairpin-helix
HIV: Human immunodeficiency virus
HNSCC: Head and neck squamous cell carcinoma
HPV: Human papillomavirus
HSV-1: Herpes simplex virus 1
IFNs: Interferons
Igfold: Immunoglobulin-like
LBR: Lamin B receptor
LCD: Low-complexity domain
LEM: LAP-Emerin-MAN1
LIHC: Liver hepatocellular carcinoma
LUAD: Lung adenocarcinoma
MMLV: Moloney murine leukemia virus
mTOR: Mammalian target of rapamycin
MTs: Microtubules
NE: Nuclear envelope
NETs: Nuclear envelope transmembrane proteins
NGPS: Néstor-Guillermo progeria syndrome
NL: Nuclear lamina
NPC: Nuclear pore complex
Nups: Nucleoporins
PARP1: Poly [ADP-ribose] polymerase 1
pBANF1: Phosphorylated BANF1
PD-1: Programmed cell death protein-1
PDAC: Pancreatic ductal adenocarcinoma
PI3K: Phosphatidylinositol 3-kinase
PIC: Pre-integration complex
PLOD3: Procollagen-lysine, 2-oxoglutarate 5-dioxygenase 3
PP2A: Protein phosphatase 2A
PP4: Protein phosphatase 4
ROS: Reactive oxygen species
SENP: Sentrin-specific protease
SF3B4: Splicing factor B subunit 4
SPTD: Strophanthidin
STING: Stimulator of interferon genes
TAD: Transactivation domain
TCD: T-cell desert
THCA: Thyroid carcinoma
TME: Tumor microenvironment
TMIGD1: Transmembrane and immunoglobulin domain-containing protein 1
TNBC: Triple-negative breast cancer
TRAIL-R2: Tumor necrosis factor -related apoptosis-inducing ligand receptor 2
Ubc9: Ubiquitin-conjugating enzyme 9
VRKs: Vaccinia-related kinases
WH: Winged-helix
